# Social Defeat Stress (SDS) in Mice: Using Swiss Mice as Resident

**DOI:** 10.21769/BioProtoc.3197

**Published:** 2019-03-20

**Authors:** Marco Oreste F. Pagliusi Jr., Cesar R. Sartori

**Affiliations:** Department of Structural and Functional Biology, State University of Campinas, Campinas, SP, Brazil

**Keywords:** Social defeat stress, Depression, Mice, Major depressive disorder, Animal model

## Abstract

**[Abstract]** Due to the high prevalence and great economic impact of depression, studies with animal models have been increasingly used to identify neurobiological mechanisms associated with this disorder. However, many animal models use stressful conditions that are not consistent with what we observe in the modern human world. Examples are the chronic unpredictable stress and the electric shock model used in rodents. It’s well established the social stress as the major cause of depressive disorder in human, in this way a social defeat stress model was recently standardized and can induce depressive-like behavior of social avoidance, a typical human depressive behavior. In this model, mice are exposed on consecutive days to an aggressor mouse, suffering brief periods of physical aggression followed by longer periods of visual and olfactory (sensory) contact and, as a consequence, a relationship of social submission is characterized. Thus, the objective of this work is to describe a social defeat stress protocol using swiss mice as resident, also describing valuable procedural suggestions that will help researchers to reproduce the model easily.

## Background

Developing animal models that mimic human psychiatric disorders is a great challenge. The difficulty is from the induction of behavioral changes associated with the psychiatric condition to the evaluation or measurement of such behavior. Depression is a psychiatric disorder with high prevalence and, as a high cognitive complexity disorder, is hard to be assessed in animal models. Stress (mainly social stress) is already well established in the literature as a key factor for the establishment of depression (Kendler *et al.*, 1999; Gold and Chrousos, 2002; Slavich and Irwin, 2014; Saleh *et al.*, 2017), so it is not surprising that animal models of depression use some kind of stress.

One of these models is the chronic unpredictable stress. In this model, rodents are exposed to a series of stressful stimuli randomly presented for several weeks. Among the stressful stimuli are: periodic deprivation of water and/or food, exposure to low temperatures, physical restraint and reversal of the light/dark cycle. As a result, the rodent exhibit a depressive-like behavior called learned helplessness, which can be reversed with antidepressant drugs. However, questions about the reproducibility of these behavioral results have been raised mainly due to the great variability of protocols, which diminished confidence in the model (Pollak *et al.*, 2010). In another animal model of depression, rodents are subjected to repeated and highly stressful electric shock sessions which generate a deficit in the aversive stimulus avoidance, a behavior typically considered as depressive. The great criticism for this model is that the depressive-like behavior does not persist after the end of electrical stimuli. These above-mentioned animal models of depression commonly use nonspecific tests to evaluate anhedonia and despair depressive-like behaviors which are commonly analyzed by the sucrose preference and forced swimming test, respectively.

In this scenario, the social defeat stress model was recently standardized and can induce depressive-like behavior of social avoidance in mice (Golden *et al.*, 2011). In this model, mice are exposed on consecutive days to an aggressor mouse, suffering brief periods of physical aggression followed by longer periods of visual and olfactory (sensory) contact. After this period a relationship of social submission is characterized, typically observed in human relations. As a result, stressed mice develop several physiological and behavioral changes, including altered levels of corticosterone, cardiac hypertrophy, circadian rhythm disturbances, widespread pain, anxiety, despair behavior, anhedonia and social avoidance (Krishnan *et al.*, 2007; Pagliusi *et al.*, 2018). Social interaction level is used to assess depressive-like behavior and, interestingly, some mice submitted to this social defeat stress protocol are resistant and do not develop social avoidance, similar to what occurs in humans, where not all individuals exposed to some form of chronic social stress develops psychopathologies (Krishnan *et al.*, 2007).

Another important feature of this model, which makes it much more reliable than the oldest animal models of depression and depressive-like behavior tests, is the fact that the social avoidance behavior can be reversed only to chronic, and not acute, treatments with antidepressant drugs, as also is observed in humans ( Berton *et al.,* 2006; Berton and Nestler, 2006; Pollak *et al.*, 2010). In addition, mice submitted to the SDS protocol exhibit neurobiological changes very similar to those found in humans with depression, including brain monoamines reduction, hypothalamic-pituitary-adrenal axis dysfunction, BDNF reduction in the hippocampus and BDNF increase in the nucleus accumbens (Hatzinger, 2000; Holsboer, 2001; Berton *et al.*, 2006; Duman and Monteggia, 2006; Krishnan *et al.*, 2007; Pittenger and Duman, 2008; Castrén and Rantamäki, 2010; Goldberg *et al.*, 2014; Iñiguez *et al.*, 2016). It is, therefore, a robust model that can induce symptoms very similar to human depression.

Thus, the objective of this work is to describe a social defeat stress protocol adapted from that described byGolden *et al.* (2011), since many laboratories can’t use the same materials and mice strain as those described in the original protocol. Therefore, we will describe how to use the Swiss mouse as an aggressor, since it is quite evident the natural aggressive behavior of this strain under the experimental conditions used in this protocol (Miczek *et al.*, 2001). We will also describe valuable procedural suggestions that will help researchers to easily reproduce the model.

## Materials and Reagents

1. Cleaning wipes
2. At least 40 Swiss mice
3. C57BL/6J mouse
4. Wood chip bedding
5. Alcohol 40% (storage at room temperature)

## Equipment

1. Timer
2. Mechanical tally counter
3. Laboratory rat breeding cage (46.3 cm [d] x 30.5 cm [w] x 15.8 cm [h]) with wood chip bedding
4. Perforated acrylic divider (43.7 cm [d] x 0.6 cm [w] x 15.8 cm [h]) that fits into the rat breeding cage and grid (see Notes)
5. Social interaction arena (42 cm [d] x 42 cm [w] x 42 cm [h]) (see Notes)
6. 1 perforated acrylic enclosure (6.5 cm [d] x 10 cm [w] x 42 cm [h]) per social interaction arena (see Notes)
7. Video tracking apparatus

## Software

1. TopScan video tracking software (Clever Sys Inc., http://cleversysinc.com/)
Consider alternative software like EthoVision^®^ XT (Noldus Information Technology, https://www.noldus.com/)
2. Prism 8.0 (GraphPad, https://www.graphpad.com/) or similar software to perform statistical analysis

## Procedure

- Swiss mice aggressive behavior screening
  1. Purchase at least 40 Swiss mice that had already performed several cycles of mating and had been retired (retired breeder). It’s necessary a large number of mice because on average only 40% are selected as good aggressors and at least 10 is required. The Swiss age should range from 6 to 12 months, which increases the percentage of mice selected as good aggressors. Prior to screening, keep them for a week single housed with free access to food and water for habituation to their new colony facility.
  2. Start screening 10 days before the initiation of the SDS sessions and, during the screening, always use the Swiss mouse home cage (with wood chip bedding). Place a C57BL/6J mouse in the Swiss home cage for 3 min (use the timer) and write down the time elapsed until the first attack (latency) and the number of attacks (use the mechanical tally counter) performed by the Swiss mouse. At this moment, the C57BL/6J is called "screener mouse" and will not be used in social defeat stress sessions. Note that the same experimenter must perform all screening sessions, using the same criteria to consider what is an attack. In order to facilitate the reproducibility, we consider attack as any aggressive action with physical contact that the Swiss mouse performs against screener mouse.
*Notes:*
    - *Stop the screening session (even before completing 3 min) if the Swiss mouse attacks the screener mouse more than 20 times, this is enough to consider the Swiss mouse as a good aggressor on that day.*
    - *If the screener mouse develops open wounds larger than 1 cm, remove it from the experiment and euthanize immediately* (Golden *et al.*, 2011).
  3. After 3 min of screening remove the screener mouse from the Swiss home cage and perform the same procedure with the other Swiss. You can use the same screener mouse with up to 5 different Swiss on the same day, otherwise, the screener mouse may become very submissive and the Swiss mouse may not attack. Therefore, to perform the screening of 40 Swiss you will need at least 8 screener mouse.
  4. Repeat Steps A2-A3 two more days (totaling 3 days of screening), each day placing a different screener mouse for each Swiss, *i.e.,* no Swiss defeats the same screener mouse twice. In this way, the Swiss mouse will not be habituated to the screener mouse.
  5. Select Swiss mice that attacked the screener mouse on at least two consecutive days with a latency less than 1 min. Discard Swiss mice that do not meet these criteria. Among those who meet these criteria, use the number of attacks to choose which Swiss mice will be used in subsequent social defeat experiments, *i.e.,* choose the Swiss mice that most attack during the 3 days of screening. Swiss mice that met the criteria but did not attack as much as the chosen ones may remain as reserves. That way, if a chosen mouse stops attacking during a defeat session you can exchange it for a reserve mouse.
  6. Following screening, single house the aggressors with free access to food and water. Before each new social defeat experiment do at least one day of rescreening, ensuring that the Swiss mice continue to behave aggressively and dominantly.
- Chronic social defeat stress
  1. Assemble the laboratory rat breeding cage (at least 10 cages–depending on how many Swiss you have selected) as shown in Figure 1, positioning the perforated acrylic divider and making sure there is free access to water and food for both sides of the cage. Always use wood chip bedding; otherwise, it will be difficult for Swiss mice to attack and for C57BL/6J to escape the attacks. At this moment the laboratory rat breeding cage will be called defeat cage.
*Note: You can perform the social defeat stress protocol with many C57BL/6J at the same time, depending only on how many residents and defeat cages you have.*  *We advise ensuring that you have at least two reserve Swiss in case you need to change some residents during defeat sessions and to use in the social interaction test (target session).*
    **Figure 1.**
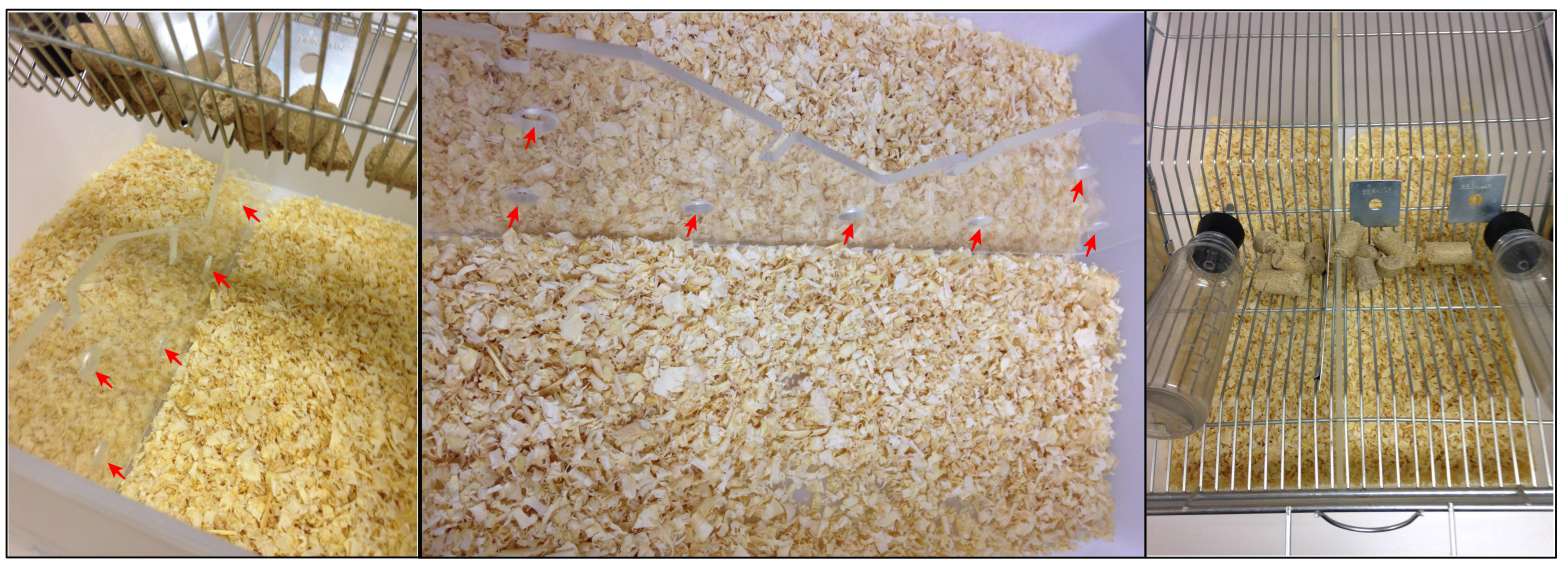
Representation of how to assemble the defeat cage. Note the perforated plastic division allowing sensory contact between mice and that both sides of the defeat cage have free access to water and food (red arrows indicate the perforations on the acrylic divider).
  2. Place one Swiss mouse, selected in Step A5, on the left side of each defeat cage. Placing them on the left side makes it easier to swap C57BL/6J mice through the defeat cages during the 10 days of social defeat. At this moment the Swiss mice will be called resident.
  3. Wait 2 days prior to start social defeat sessions. It is necessary for the habituation of the resident mouse to the new environment.
  4. Place one C57BL/6J mouse on the left side of each defeat cage (same side of the resident mouse). After 10 min of social defeat stress, place the C57BL/6J on the right side of the same defeat cage, separated from the resident by the perforated acrylic divider. Now they are physically separated but still have sensory contact. Assemble more defeat cages to place C57BL/6J controls. Control mice will have no social contact during the 10 days of social defeat. They will be continuously separated by the perforated acrylic divider paired with another C57BL/6J control mouse.
  5. On the next day (preferably at the same hour as the previous day), place the defeat cages side by side on a bench or table. Take the C57BL/6J mouse from the first defeat cage and place it on the left side (same side of the resident mouse) of the next defeat cage. Perform this procedure with all defeat cages, placing the last C57BL/6J mouse on the left side of the first defeat cage (see Figure 2). After 10 min of social defeat, separate the mice by placing the C57BL/6J on the right side of the defeat cage. Rotate the C57BL/6J control mice in a way that they are not paired with the same control mouse from the previous day.
  6. Repeat Step B5 another 8 times, totaling 10 days of social defeat stress. On the last day of social defeat put the C57BL/6J mice in a standard mouse cage with free access to water and food (single housed). On the next day, perform the social interaction test.
*Notes:*
    - *As already mentioned, the resident mouse may not attack the C57BL/6J mouse in some social defeat stress session. In this case, you can exchange it for one of the previously selected reserve Swiss mice (Step A5). In our hands, this is rare to happen since over the course of the defeats the resident mice become more aggressive. In some cases, the C57BL/6J mouse becomes very submissive, and the resident mouse may not attack. In such a case, it is not advisable to change the resident.*
    - *C57BL/6J mice may be injured during the 10 days of social defeat stress, especially in the posterior dorsal region. In these cases, you can treat the wound with antiseptic spray at the end of each social defeat stress session. If the injury becomes too severe (open wounds larger than 1 cm) remove the animal from the experiment and euthanize immediately*.
  **Figure 2.**
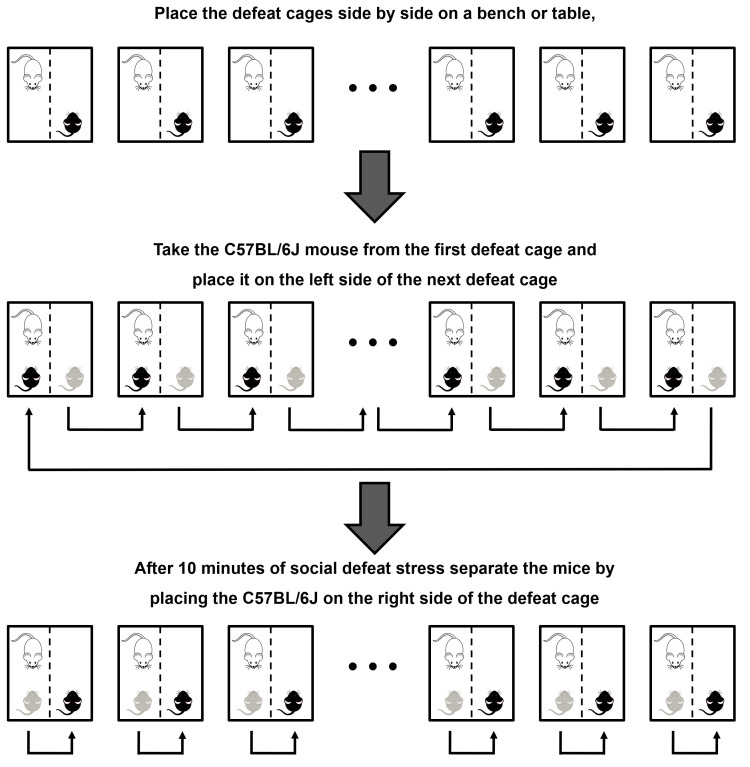
Graphic representation of how to perform the rotation of the C57BL/6J–black–mice from the second day of social defeat stress. Place the defeat cages side by side on a bench or table. Take the C57BL/6J mouse from the first defeat cage and place it on the left side (which contain the resident-white-mouse) of the next defeat cage. Perform this procedure with all defeat cages, placing the last C57BL/6J mouse on the left side of the first defeat cage. After 10 min of agonistic contact (social defeat), separate the mice by placing the C57BL/6J on the right side of the same defeat cage. On the tenth day, instead of separating side-by-side the animals at the end of 10 min of social defeat stress, put the C57BL/6J mice in their respective home cages (single housed) with free access to food and water.
- Social interaction test
  1. Place the social interaction arena under the video tracking apparatus, in a way that the whole arena is being recorded in a top view. Clean the arena with 40% alcohol, which will clean the surface and draw odors. Note that the arena has two regions of interest: the interaction zone and the corners. The size and layout of each zone are shown in Figure 3.
*Note: Interaction zone and corner markings in the social interaction arena can be created with the video tracking software. Make sure that the interaction arena has a white floor so the software easily recognizes the contrast with the black mouse (C57BL/6J).*
    **Figure 3.**
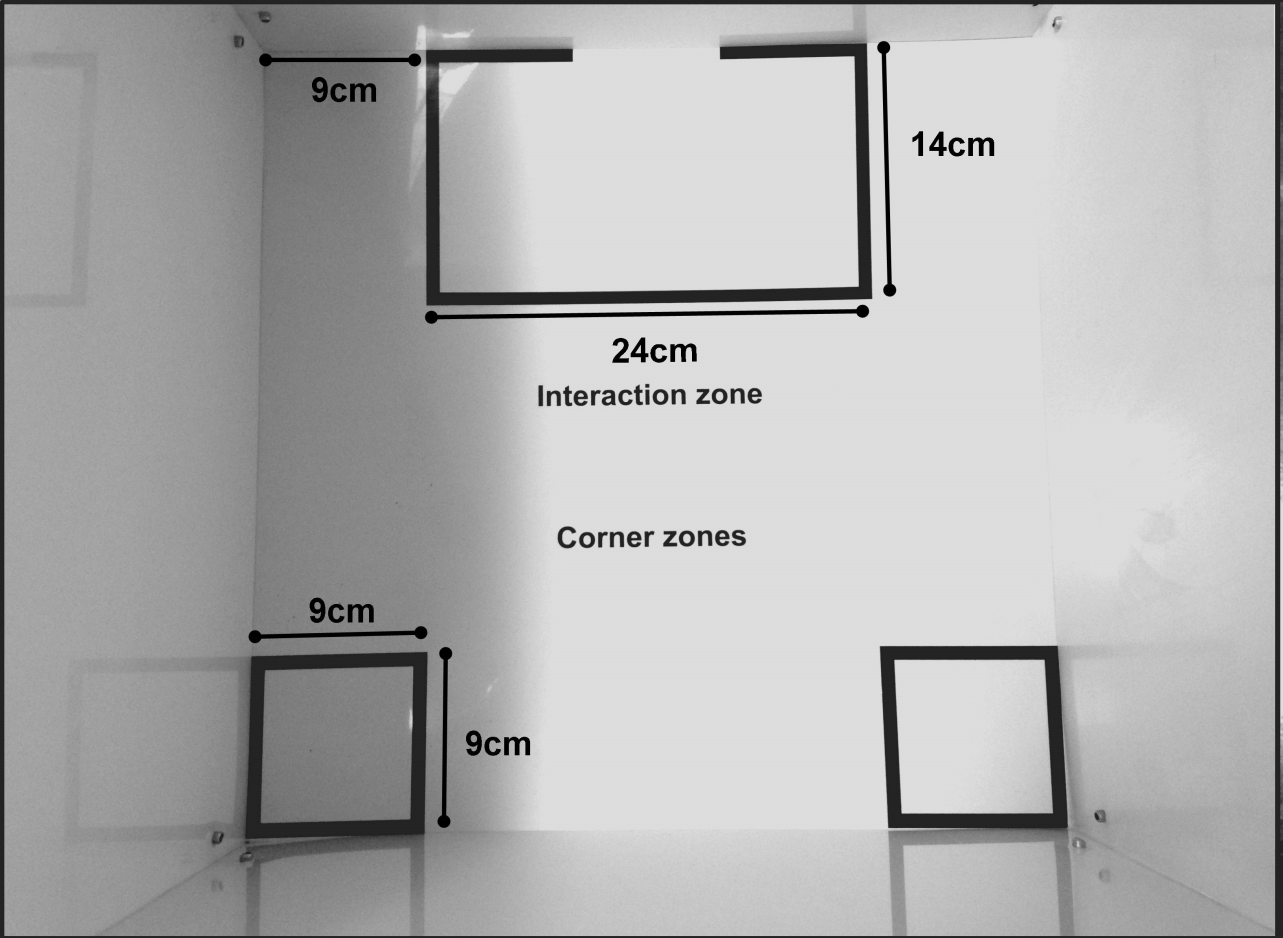
Top view of the open field arena used in social interaction test showing the measures and layout of the interaction zone and corners
  2. Place an empty perforated acrylic enclosure in the center of the interaction zone as shown in Figure 4.
    **Figure 4.**
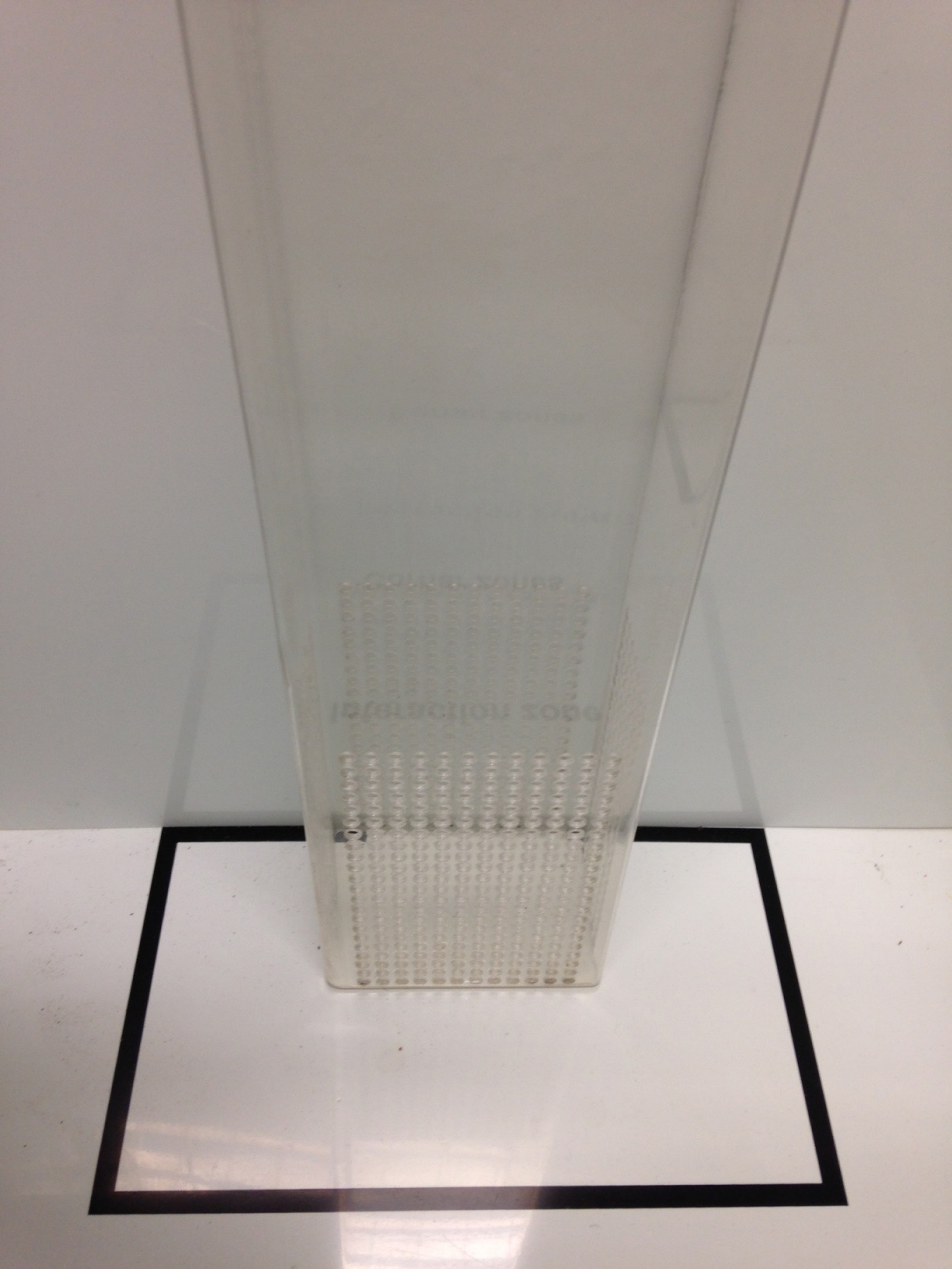
Representation of how to place the perforated acrylic enclosure. The black lines are delimiting the interaction zone.
  3. Place the C57BL/6J into the rear part of the arena opposite to the interaction zone. Record the session for 150 s. This first session is called "no-target" because the perforated acrylic enclosure is empty.
  4. Remove the C57BL/6J and place in his home cage. Place an unknown aggressor Swiss mouse (who has not participated in the aggression sessions–you can use a reserve Swiss) inside the perforated acrylic enclosure.
  5. Wait for 30 s from the Step C3 and then place the same C57BL/6J into the rear part of the arena opposite the interaction zone. Record the session for 150 s. This second session is called "target" because the perforated acrylic enclosure contains an aggressive Swiss mouse.
  6. Remove the C57BL/6J and the Swiss place them in their respective home cage. Clean the arena and the perforated acrylic enclosure with 40% ethanol.
  7. Repeat Steps C2-C6 with all C57BL/6J mice, including controls.

## Data analysis

**Figure 5.**
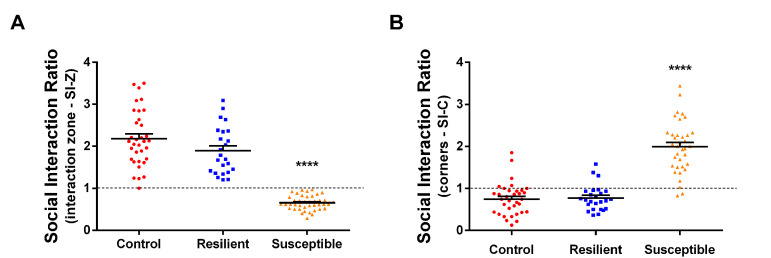
Social interaction ratio. A. Social interaction ratio in the interaction zone (SI-Z) calculated as the ratio between the time in the interaction zone in the target session divided by the time in the interaction zone in the no-target session. This ratio is used to separate the stressed mice between susceptible and resilient groups, where stressed mice with SI-Z < 1 are considered susceptible and stressed mice with SI-Z ≥ 1 are considered resilient. B. Social interaction ratio in the corners (SI-C) calculated as the ratio between the time in the corners in the target session divided by the time in the corners in the no-target session. (n = 24-41; ****P < 0.0001, statistical difference).

Using a tracking software (such as TopScan-CleverSys Inc.) analyze the videos and get the time spent in interaction and corners zones in both sessions: target and no-target. If you do not have the appropriate software, you can analyze the video and measure the time in each zone manually using the timer. With these data you can obtain the social interaction ratio in the interaction zone (SI-Z) which is calculated by dividing the time spent in the interaction zone during the target session by the time spent in the interaction zone during the no-target session. The SI-Z ratio equal to 1 is used as the threshold to separate stressed mice into the susceptible and resilient groups. Mice presenting SI-Z lower than 1 are classified as susceptible, *i.e.,* show depressive-like behavior of social avoidance (see Figure 5A). On the other hand, mice presenting SI-Z equal or higher than 1 are classified as resilient, *i.e.,* even submitted to the social defeat stress they don't show depressive-like behavior of social avoidance (see Figure 5A). Also, it is possible to obtain the social interaction ratio in the corners (SI-C) which is calculated by dividing the time spent in the corners in the target session by the time spent in the corners in the no-target session (see Figure 5B). SI-C higher than 1 means that mice spent more time avoiding the interaction zone in the “target” session, which can corroborate the SI-Z data. Our experience with the model shows that 20% to 40% of stressed mice are expected to become resilient, which means that up to 80% of the stressed mice show depressive-like behavior of social avoidance. It is important to mention that the depressive-like behavior of social avoidance induced by the SDS model can last up to 4 weeks (our group did not test a longer period than this), which means that these mice can be used for chronic experiments without problems.

**Figure 6.**
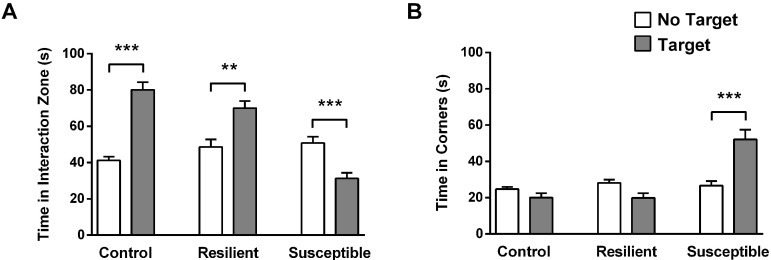
Total time spent in interaction and corners zones. A. Total time spent by each group (resilient and susceptible were separated by SI-Z) in the interaction zone in the target and no-target sessions. B. Total time spent by each group in the corners in the target and no-target sessions (n = 24-41; **P < 0.01; ***P < 0.001 statistical difference).

The results can also be shown using the total time spent in the interaction zone and corners (see Figure 6), although only the SI-Z is used to separate between resilient and susceptible. Data should be analyzed in specialized software (such as Prism 8.0-GraphPad) capable of performing one-way analysis of variance (ANOVA) followed by Bonferroni multiple comparison tests, comparing the SI-Z, SI-C or the total time of the controls, resilient and susceptible. The results should be considered statistically relevant when *P* < 0.05.

## Notes

**Figure 7.**
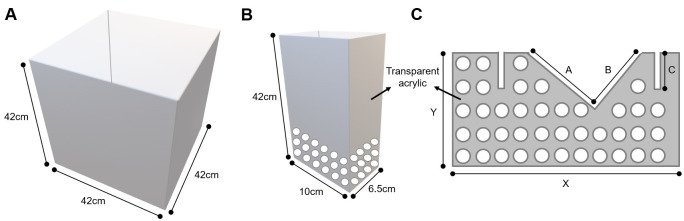
Detailed design of the apparatus. A. Measures for social interaction arena (build the arena with an easy-to-clean material such as epoxy or acrylic). B. Measures for perforated transparent acrylic enclosure (perforations should be no more than 1 cm in diameter). C. Measures for perforated transparent acrylic divider (X and Y depend on the size of your rat breeding cage and A, B and C depend on the size of your grid that covers the rat breeding cage–perforations should be no more than 1 cm in diameter).

Perforated acrylic divider and enclosures as well as social interaction arena can be obtained from any plastic and Plexiglas local vendor. Show the manufacturer your own rat breeding cage and the measures of the enclosures and social interaction arena shown in this protocol (Figure 7); they should know how to construct the apparatus.
